# Outcome of Patients with NSCLC and Brain Metastases Treated with Immune Checkpoint Inhibitors in a ‘Real-Life’ Setting

**DOI:** 10.3390/cancers12123707

**Published:** 2020-12-10

**Authors:** Marcus Skribek, Konstantinos Rounis, Dimitrios Makrakis, Sofia Agelaki, Dimitris Mavroudis, Luigi De Petris, Simon Ekman, Georgios Tsakonas

**Affiliations:** 1 Thoracic Oncology Center, Theme Cancer, Karolinska University Hospital Section, Karolinska University Hospital, 17164 Stockholm, Sweden; marcus.skribek@ki.se (M.S.); kostas@rounis.gr (K.R.); luigi.depetris@ki.se (L.D.P.); georgios.tsakonas@ki.se (G.T.); 2 Department of Oncology-Pathology, Karolinska Institutet, 17164 Stockholm, Sweden; 3 Department of Medical Oncology, University General Hospital, 71110 Heraklion, Greece; dim.ph.makrakis@gmail.com (D.M.); agelakisofia@gmail.com (S.A.); mavrudis@med.uoc.gr (D.M.); 4 Division of Oncology, University of Washington Medical School, Seattle, WA 98195, USA

**Keywords:** immunotherapy, immune checkpoint inhibitors, advanced lung cancer, non-small cell lung cancer, brain metastases

## Abstract

**Simple Summary:**

We identified non-small cell lung cancer (NSCLC) patients with brain metastases who were treated with immune checkpoint inhibitors at Karolinska University Hospital, Sweden and University Hospital of Heraklion, Greece from 2016 to 2019. We analyzed intracranial efficacies in the patients who had not received local treatment for their brain metastases less than three months prior to the initiation of immune checkpoint inhibitors and had adequate radiological evaluation. We demonstrated that immune checkpoint inhibitors are active in NSCLC patients with brain metastases regardless of the presence of neurological symptoms. This is a novel finding since, until now, this patient group has irrefutably been underrepresented in clinical studies and there is a clear scarcity of data. The results of our analyses suggest that symptomatic patients with active brain metastases (BM) may be considered for immunotherapy in routine clinical practice as well as clinical trials.

**Abstract:**

There is lack of data addressing the intracranial (IC) efficacy of immune checkpoint inhibitors (ICIs) on brain metastases (BM) in non-small cell lung cancer (NSCLC). This patient category is underrepresented in randomized clinical trials. We retrospectively collected clinical data on patients with non-oncogenic driven NSCLC with BM who were treated with ICIs at two medical oncology institutes in Sweden and Greece from 2016 to 2019. IC efficacy was assessed in patients who had not received local treatment for BM less than three months prior to the initiation of ICIs and had adequate radiological evaluation. We screened 280 patients, of which 51 had BM. BM was an independent predictor for inferior PFS (HR = 2.27; 95% CI, 1.53–3.36) but not OS (HR = 1.58; 95% CI, 0.97–2.60) for the whole patient population. IC response assessment was done on 33 patients. IC objective response rate (ORR) was 24.2%. The presence of neurological symptoms related to BM did not affect IC ORR (*p* = 0.48). High PD-L1 levels from extracranial biopsies were not a predictive factor for IC ORR (*p* = 0.13). ICIs are active in NSCLC patients with BM regardless of the presence of neurological symptoms and can achieve durable IC disease stabilization in a subgroup of patients.

## 1. Introduction

Despite recent advances in diagnosis and treatment of non-small cell lung cancer (NSCLC), prognosis remains poor with five-year survival rates of around 5% in the metastatic setting [1]. The presence of brain metastases (BM) in NSCLC patients is an adverse prognostic factor, with increased incidence in adenocarcinoma and tumors harboring oncogenic driver mutations [2]. Approximately 25% of NSCLC patients present with BM at diagnosis [3].

The treatment for NSCLC with BM consists of two modalities; local treatment for CNS disease and systemic treatment [4,5,6,7]. The efficacy of targeted therapies in patients with oncogenic driven NSCLC with BM has been demonstrated in several studies, resulting in high intracranial (IC) objective response rates (ORR) and prolonged survival [8,9,10]. Conversely, for patients without oncogenic drivers, systemic chemotherapy with a platinum backbone has been the accepted standard-of-care for two decades, yielding an IC ORR in the range of 20–40% [7]. Survival outcomes after systemic chemotherapy have been modest, with real-world data demonstrating median overall survival (OS) times ranging from 5.6 months to 9.3 months [11].

The development of immune checkpoint inhibitors (ICIs) has paved a new era of cancer treatment. The use of anti-PD-1/PD-L1 antibodies in the second-line setting is well documented as monotherapy, where a longer OS was observed in comparison to standard docetaxel [12,13,14]. Several recent first-line studies with ICIs alone or in combination with chemotherapy represent the new standard-of-care [15,16,17,18,19]. However, the use of ICIs in NSCLC patients with BM is a matter of ongoing debate due to the scarcity of available data and the concerns about immunotherapy efficacy in the context of the divergent tumor microenviroment (TME) in the CNS [20,21].

Historically, the CNS has long been considered to be an immune-privileged site. This central dogma has recently been challenged due to the discovery of CNS lymphatics and is now considered to be immune-competent [22]. The blood-brain barrier is believed to be a relative rather than an absolute barrier, allowing the permeation of immune cells and systemic therapies into the CNS [23]. A number of studies have highlighted the potential of ICIs to alter the immune context of the CNS, leading to effective IC antitumor response [24,25,26,27].

However, the pivotal clinical trials that lead to the approval of ICIs for NSCLC have only included patients with stable BM; active BM were part of the exclusion criteria. Therefore, these trials examined the IC efficacy of ICI treatment in a selected patient population [12,13,14,15,16,17]. KEYNOTE-024 was a randomized phase III trial investigating the activity of pembrolizumab versus chemotherapy in the first-line setting for advanced NSCLC patients with PD-L1 expression ≥ 50% [28]. In this trial, 11.8% of the patients who received first-line pembrolizumab had baseline BM and this subgroup exhibited an inferior progression-free survival (PFS) compared to those without BM [28]. In the phase 3 OAK trial where atezolizumab was compared to docetaxel in the second line setting, a subgroup analysis that consisted of patients with asymptomatic BM showed that individuals who received atezolizumab as a second-line treatment exhibited a longer OS than those treated with docetaxel [13]. Moreover, a recent single cohort phase 2 trial examined the efficacy of pembrolizumab monotherapy in patients with BM from NSLCL and reported an IC ORR of 29.7% [29]. However, these patients included in the study by Goldberg et al. [29] had to have asymptomatic BM, absence of corticosteroid requirements, and were permitted to have received cranial irradiation until two weeks before the initiation of pembrolizumab monotherapy. Finally, a recently published retrospective analysis evaluated the efficacy of ICIs in 255 patients who had metastatic BM due to NSCLC before ICI therapy initiation. Of these, 73 patients had active BM that were defined as newly diagnosed and non-irradiated lesions and/or growing lesions on brain imaging without subsequent local treatment before the start of ICI treatment. Only 12% of the patients with active BM exhibited neurological symptoms attributed to their CNS metastases at the time-point of ICI administration. This study reported an IC ORR of 27.3% in the patients with active BM and the median OS was 8.6 months versus 11.4 months for patients with and without BM, respectively [30].

BM as a consequence of NSCLC has irrefutably been underrepresented in clinical studies with ICIs and there is a scarcity of data addressing their effect in the real-world setting. To this end we have conducted a ‘real-life’, multicenter, retrospective observational study to shed further light on this topic. We included patients with both stable and unstable baseline BM and patients with neurological deficits attributed to their BM prior to ICI therapy in order to examine its effects on response and survival outcomes.

## 2. Results

### 2.1. Patient Characteristics

A total of 280 patients were analyzed, of which 51 patients had brain metastases. Baseline patient characteristics are summarized in Table 1. In the brain metastatic patient group, median age was 69 years (range: 40–84 years), performance status (PS) was 0–1 in 74.5% of the patients, CNS symptoms associated with the presence of BM were seen in 22 patients (43.1%) and 42 (84.2%) of our patients were classified as having active BM.

Of the 51 brain metastatic patients, 33 fulfilled the criteria for response assessment. Out of these 33 patients, 25 were categorized as having active BM. Furthermore, 10 patients received ICIs as first-line therapy and 41 patients received ICIs as second or subsequent line. Primary BM was identified in 31 patients. Radiologic evaluation was also analyzed, where 60.8% had an MRI to evaluate IC response and 39.2% had a CT scan. Previous local CNS treatment modalities prior to the initiation of ICI are demonstrated in Figure S1.

### 2.2. Response Assessment

**Figure 1:**
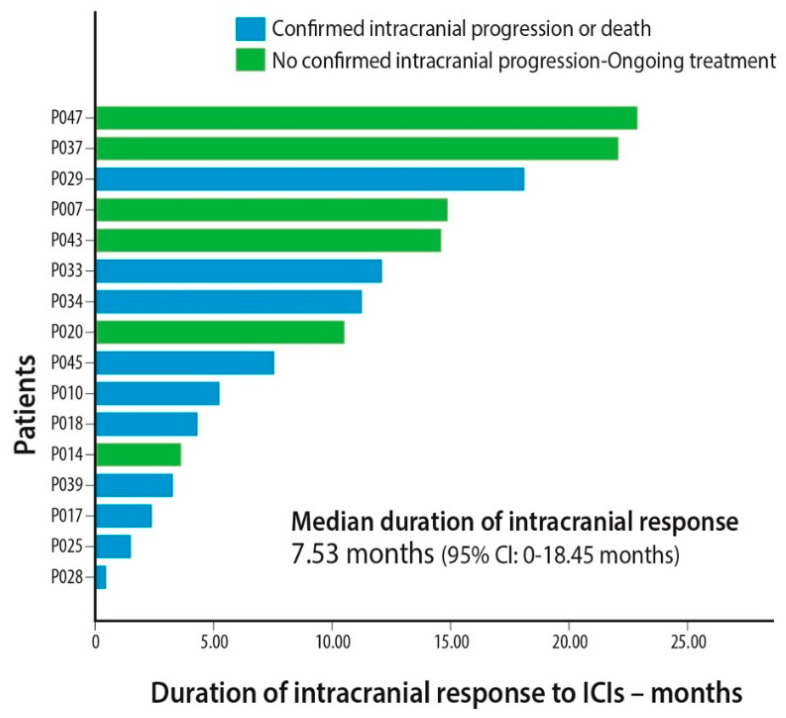
Intracranial duration of response for each patient that achieved partial response or disease stabilization in CNS lesions. Abbreviations: ICIs = Immune Checkpoint Inhibitors, 95% CI = 95% Confidence Interval.

A total of 33 patients fulfilled the pre-specified criteria for IC response assessment. In their first radiological assessment after ICI administration, one (3%) had complete IC remission (CRi), seven (21.2%) had partial remission in their BM (PRi), eight (24.2%) had IC stable disease (SDi), and 17 (51.5%) experienced progressive IC disease (PDi) (Table 2). Patients who achieved PRi or SDi in their BM experienced a prolonged IC remission (Figure 1). Median duration of IC response was 7.5 months (95% CI, 0.00–18.45). In the subgroup of 25 patients with active BM, IC ORR was 24% (one CRi and four PRi), 8% had SDi and 68% experienced PDi (Table 2).

**Figure 2:**
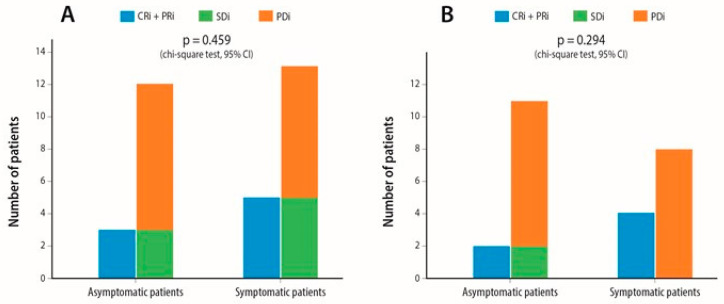
Association between presence of symptoms attributed to brain metastases and intracranial overall response rate; p-value of overall response rate (blue bars) between symptomatic and asymptomatic patients are shown in each graph (**A**) In the whole patient population that was included for response assessment (**B**) In the subgroup of patients with active brain metastases. Abbreviations: 95% CI = 95% Confidence Interval, CRi = Intracranial Complete Response, PRi = Intracranial Partial Response, SDi = Intracranial Stable Disease, PDi = Intracranial Progressive Disease.

The presence of neurological symptoms associated with BM did not affect IC ORR when compared to asymptomatic patients (*p* = 0.46) (Figure 2A). Patients who received steroids of >10 mg prednisolone equivalent for ≥10 days did not experience inferior IC ORR compared to those who were steroid naïve (*p* = 0.62) (Figure S2A). In addition, high PD-L1 expression (≥50%) levels extracranially did not constitute a positive predictive factor for superior IC ORR (*p* = 0.13) (Figure S3A). The same pattern of IC ORR was observed amongst the subgroup of patients with active BM. Symptoms due to BM (*p* = 0.29), corticosteroid requirements (*p* = 0.91) and extracranial PD-L1 expression levels ≥ 50% (*p* = 0.18) did not affect IC ORR in patients with active BM (Figure 2B, Figures S2B and S3B). Median IC TTP was 2.5 months (95% CI, 1.45–3.35).

**Figure 3:**
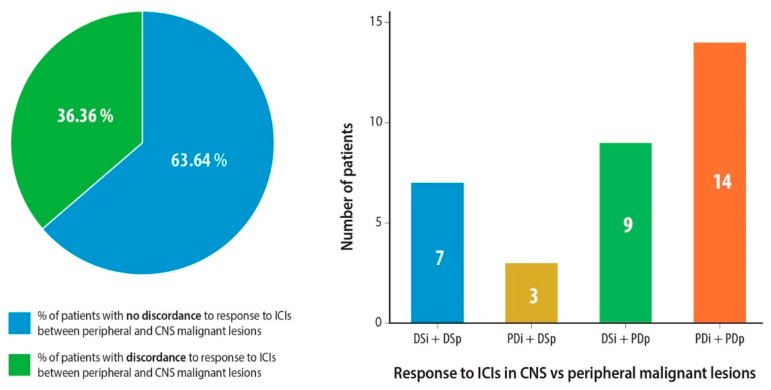
Discordance in disease stabilization (partial response or stable disease) after ICI administration between CNS and peripheral lesions. Abbreviations: ICIs = Immune Checkpoint Inhibitors, DSp = Peripheral Disease Stabilization, PDi = Intracranial Disease Progression, DSi = Intracranial Disease Stabilization, PDp = Peripheral Progressive Disease.

Interestingly, we found a high discordance rate between disease stabilization rates (PR or SD) in the extracranial versus CNS lesions at a level of 36.3%. A total of 27.2% experienced disease stabilization in the CNS and progressive disease in the extracranial metastatic sites (Figure 3). In patients with active BMs, six out of 25 (24%) had discordance between IC and extracranial disease stabilization rates (Table S1). Three patients (9.1%) had the opposite with DSp and PDi.

### 2.3. Survival Outcomes

**Figure 4:**
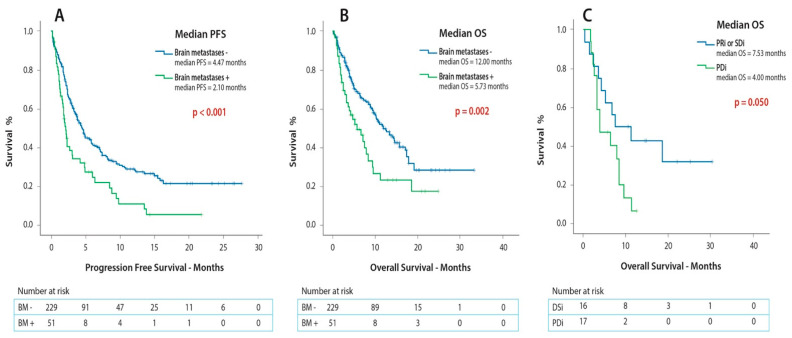
Kaplan-Meier curves on the effect of (**A**) Presence of brain metastases on PFS (**B**) Presence of brain metastases on OS (**C**) Achieving intracranial disease stabilization as a first response to ICI administration on OS. Abbreviations: ICI = Immune Checkpoint Inhibitor, PFS = Progression-Free Survival, OS = Overall Survival, PRi = Intracranial Partial Response, SDi = Intracranial Stable Disease, PDi = Intracranial Progressive Disease.

In the initial study population (*n* = 280), the presence of BM was associated with inferior PFS (1.9 versus 4.5 months; *p* < 0.001) and OS (5.7 versus 12.0 months; *p* = 0.002) in comparison to the patients without BM (Figure 4A,B). The results of the survival analyses showed that the presence of BM independently predicted for inferior PFS (HR = 2.27; 95% CI, 1.53–3.36) but not for OS (HR = 1.58; 95% CI, 0.97–2.60) (Table 3).

Amongst the patients with BM (*n* = 51), the presence of neurological symptoms did not affect PFS (1.6 versus 2.2 months; *p* = 0.40) or OS (3.6 versus 7.5 months; *p* = 0.88) (Figure S4A,B). Out of all the other studied covariates, the achievement of IC disease stabilization (PRi or SDi) was associated with superior OS (7.5 versus 4.0 months; *p* = 0.05) (Figure 4C) and a high disease burden was correlated with inferior survival (3.6 versus 8.5 months; *p* = 0.02) in the patients with BM (Figure S5). Furthermore, a high disease burden constituted the only independent predictor for shorter OS (HR = 2.39; 95% CI, 1.16–4.92) in patients with BM (Table 4). In addition, none of the analyzed covariates independently predicted for inferior IC TTP (Table S2).

PFS and OS for patients with BM (*n* = 51) was 1.9 months (95% CI, 1.53–2.33) and 5.7 months (95% CI, 2.58–8.88), respectively (Table 2).

## 3. Discussion

In this ‘real-life’, multicenter, retrospective observational study patients with symptomatic and active BM were included. The cut-off for previous local CNS treatment was set at more than three months for IC response assessment in order to examine the actual effect of ICIs in BM. This study has found that ICIs are active in these patients regardless of the presence of neurological symptoms.

ICI administration exhibited an IC ORR of 24.2% in the 33 patients that fulfilled the criteria for IC response and an IC ORR of 24% in the subgroup of 25 patients with active BM. A meaningful clinical benefit (31.3%) was observed amongst the 16 patients who achieved IC disease stabilization (PRi or SDi). This clinical benefit is further demonstrated by the prolonged duration of IC remission. Our data are in accordance with the data published by Goldberg et al. and Hendriks et al. [29,30], who reported similar IC ORR and confirmed that ICIs are active in the treatment of BM from NSCLC. Due to the pre-specified cut-off of a more than three-month interval between the last administration of local treatment for BM and ICI initiation, the ORR that is presented in our cohort can be attributed mainly to the effect of ICIs in the BM rather than to a delayed or prolonged response to prior IC radiation. Whereas our results are clinically significant in terms of ORR, they are analyzed from a selected patient group and translational research should be done to identify potential biomarkers to explain these findings.

In addition, 43.1% of our patients experienced neurological symptoms associated with the presence of BM in contrast with Goldberg et al. [29] where symptoms associated to BM were part of the exclusion criteria and Hendriks et al. [30] where only 12% of patients with active BM had symptoms. Our study presents novel data that ICI administration is active against BM in symptomatic patients. The presence of neurological symptoms did not affect IC ORR, IC TTP, PFS or OS. The results of our analyses suggest that symptomatic patients with active BM should be considered for inclusion in clinical routine as well as for inclusion in future clinical trials.

Intriguingly, we found a high discordance rate of 36.3% between IC and extracranial disease stabilization rates. Our discordance rates are slightly higher than those reported by Goldberg et al. (22%) [29] and Hendriks et al. (12.7%) [30], something which can be attributed to a selection bias in these two trials. In the trial by Hendriks et al. [30], ORR was analyzed, but not disease stabilization rate and it consisted of a mixed cohort (patients from randomized prospective clinical trials, real-world data, and had received local CNS therapy). In our cohort, the discordance rate in patients with active BMs was 24%, which was closer to the rates reported in the two above-mentioned trials. Comparative gene expression and tissue analyses of matched extracranial and BM samples of the same patients have demonstrated that the TME in BM is characterized by increased expression of molecular markers attributed to a high density of M2 macrophages, reduced CD8+ T cell infiltration [20] and downregulation of gene expression of *CCL19* and *CCL21*, which are responsible for T cell chemotaxis [21]. These significant differences in the TME context between the CNS and the extracranial tissues justify our findings, but further research at a clinical and molecular level is required.

High PD-L1 expression of ≥50% in extracranial biopsy samples was not associated with increased IC ORR. Despite being the only approved biomarker for clinical decision making, PD-L1 status may not sufficiently reflect the immune status of the CNS metastases due to the inherent differences in brain and extracranial tissue [20,21]. Therefore, in a patient with high PD-L1 expression levels on a biopsy sample from an extracranial tissue, we should not solely rely on ICIs as a single agent for the patient’s treatment plan; physicians should exploit other treatment options with either systemic (combining chemotherapy-immunotherapy) or local treatment modalities (surgery, SRS). Larger data analysis is needed to further test this hypothesis.

Moreover, the presence of BM was an independent predictor for inferior PFS but not OS in comparison to the patients without CNS dissemination and these findings are in accordance with similar studies [30]. However, the design of future clinical trials including symptomatic patients with active BM is recommended.

Finally, the only parameter that was associated with reduced OS in our cohort was the presence of metastatic disease in more than two organs, probably reflecting a more aggressive underlying disease biology. Interestingly, it has been the only parameter thus far associated with an increased risk in developing a hyperprogressive disease after administration of ICIs as a single agent [31].

To our knowledge this is one of the few studies examining real-world data on the effect of ICIs in metastatic NSCLC patients with BM. Our population samples consist of a high percentage of patients with symptomatic and active BM as opposed to previous publications. Furthermore, it provides a discreet methodology in order to avoid a potential confounding factor between the effect of systemic ICI treatment and the potential lasting effect of local treatment modalities due to the pre-specified three-month window that was set before the initiation of ICIs.

The limitations of the study include the retrospective design, a relatively small patient population with limited statistical power, and the heterogeneity of the studied population, as patients received ICIs in different line settings. To confirm whether ICIs in the CNS are in fact effective and the various parameters that potentially affect patients’ responsiveness and survival outcomes, larger prospective studies should be carried out and evaluated to further validate the results of our trial.

## 4. Methods

### 4.1. Patient Population/Study Design

We initially screened a total of 280 patients, 197 patients from Karolinska University Hospital and 83 patients from University Hospital of Heraklion who received ICIs in the setting of metastatic NSCLC, between December 2016 and February 2020. Patients with BM due to NSCLC, without clinically actionable mutations, and received at least one dose of ICI were included in the survival analysis. The ICIs used were atezolizumab, nivolumab and pembrolizumab. All treatments were administered with standard doses and schedules according to international guidelines specific to the tumor type and according to the treating physician. Our study was reviewed and approved by the national ethical institutional review board (DNR 2020-02636).

For the assessment of IC response to immunotherapy, we further excluded patients who had received ICIs in combination with chemotherapy, combination of anti-PD-1/PD-L1 agents with anti-CTLA-4 antibodies, those who had received local treatment modalities (surgery, SRS, WBRT) within three months before the initiation of ICIs or after their first administration and patients who did not have subsequent CNS radiological evaluation addressing IC response to ICIs (Figure S6).

The following covariates were analyzed: age, gender, smoking status, PS, tumor histology, extracranial PD-L1 expression, number of organs with metastatic disease, presence of neurological symptoms attributed to BM, local CNS treatment modality (WBRT, SRS, surgery), line of treatment of ICI administration, presence of active BM, baseline steroid administration with >10 mg prednisolone equivalent for ≥10 days, ICI start/end-date and date of death or last follow-up. The clinical stages were characterized according to the 8th edition of the TNM classification.

Active BM were defined as IC lesions not previously treated or progressing after previous radiotherapy, systemic chemotherapy or surgical excision. We further subcategorized the patients based on the number of organs affected by metastatic disease. Specifically, high disease burden was defined as more than two organs affected by metastatic disease (including BM or lung metastases but excluding lymph node metastases). The rationale for this cut-off is based on the publication by Ferrara et al., demonstrating that metastatic disease with more than two organs was associated with the development of hyperprogressive disease [31]. In addition, an individual was categorized as having primary CNS disease if BM were present at diagnosis and as non-primary CNS disease if the patient had developed CNS lesions later during the disease trajectory but prior to the initiation of immunotherapy.

### 4.2. Outcome Assessment

Response evaluation was performed for IC and extracranial lesions in the first radiologic assessment after ICI administration and after each follow-up [32]. Duration of IC response was calculated as the time from the initiation of ICI to the date of IC progression or death after data censoring amongst the patients who achieved IC partial response or IC stable disease according to RECIST 1.1 criteria [32].

PFS was defined as the time from the initiation of ICI to the date of disease progression or death. OS was defined as the time from the start of ICI to death. IC time to progression (TTP) was defined as the time from the initiation of ICI to the date of radiologically confirmed progression by brain CT or MRI scan or the date of death. Patients who were still alive or had not progressed were censored at the date of last contact.

### 4.3. Statistical Analyses

Descriptive statistics were performed to analyze categorical and continuous variables. Kaplan–Meier curves were plotted to determine OS, PFS and IC TTP. Patients who were alive at the time of data collection were treated as censored observations during the analysis. Curves were compared with the log-rank test. The chi-squared test was used to assess statistical heterogeneity between categorical variables. A *p*-value of ≤0.05 (two-sided test) was considered statistically significant.

Cox proportional regression was performed for univariate and multivariate analyses to estimate the hazard ratios (HR) together with the 95% confidence intervals (CI). We performed a univariate analysis for the patients with BM, addressing the effect of the following parameters on OS: age ≥ 70 years old, PS, histology, line of treatment when ICI was administered, high disease burden, non-primary BM and PD-L1 expression levels ≥ 50% extracranially. We then performed a multivariate analysis including the variables that had reached statistical significance in the univariate analysis. All statistical analyses were performed using the SPSS 25.00 software (IBM Corp., Armonk, NY, USA).

## 5. Conclusions

This study demonstrated that ICIs are active in NSCLC patients with BM, irrespective of the presence of neurological symptoms due to their CNS metastases, and these patients should not be excluded from future clinical trials. Moreover, PD-L1 status extracranially was not predictive of IC ORR or outcome in our study and further research is warranted to further analyze the role of PD-L1 expression in patients with BM due to the different context of the TME between the brain and the extracranial tissues.

## Figures and Tables

**Table 1:** Patient characteristics.

Patient Characteristics		Total Patients with Brain Metastases (*n* = 51)	Total Patient Population (*n* = 280)
Age (years)	Median	69	69.8
	Range	40–84	35–84
Gender (male)		54.9%	55.7%
Performance status 0–1		74.5%	83.5%
Smoking status (former or active)		86.3%	92.8%
Histology (non-squamous)		84.3%	67%
PD-L1 expression extracranially	<1%	7.8%	10%
	1%≤PD-L1<50%	25.5%	28.9%
	PD-L1≥50%	58.5%	34.6%
	No data	19.6%	26%
Line of treatment of ICI administration (first line)		19.6%	21%
Types of ICI Therapy Administered	Atezolizumab	3.9%	
	Nivolumab	51.0%	
	Pembrolizumab	45.1%	
Mean number of ICI cycles		6.2 (range: 1–42)	
Primary CNS metastases at diagnosis		60.8%	
Neurological symptoms associated with CNS metastases		43.1%	
Radiological evaluation method of CNS metastases (MRI)		60.8%	
Size of largest CNS metastasis (mm)	Median	17	
	Range	4–50	
> 3 CNS metastases		33.3%	
High disease burden *		31.4%	
Steroid administration > 10 mg for ≥10 days		54.1%	
Active brain metastases		84.2%	
Previous local CNS treatment modality	WBRT	33.3%	
	Surgical excision	5.9%	
	SRS	29.4%	
	Surgical excision + SRS	2.0%	
	WBRT + SRS	2.0%	
	No local treatment	27.5%	
Timeline of local CNS treatment modality administration	≥3 months before ICI administration	33.3%	
	<3 months before or after ICI administration	39.2%	
	No local treatment	27.4%	
	Range	0.4–22.9	

Abbreviations: ICIs = Immune Checkpoint Inhibitors, WBRT = Whole Brain Radiotherapy, SRS = Stereotactic Radiosurgery. * High disease burden is defined as >2 organs affected by metastatic disease.

**Table 2:** Patient outcomes.

Patient Outcomes		Total (*n* = 33)	Total (*n* = 51)	95% Confidence Intervals
Intracranial response rates to ICIs	CRi	3.0%		
	PRi	21.2%		
	SDi	24.2%		
	PDi	51.5%		
Intracranial response rates to ICIs in patients with active brain metastases	CRi	4.0%		
	PRi	20.0%		
	SDi	8.0%		
	PDi	68.0%		
Duration of intracranial response (months) (patients that achieved PRi or SDi)	Median	7.5		0.0–18.5
	Range	0.5–22.9		
Disease progression			88.2%	
Death			78.4%	
Time to intracranial progression (months)	Median		2.5	1.5–3.6
	Range		0.2–22.9	
PFS (months)	Median		1.9	1.5–2.3
	Range		0.1–22.1	
OS (months)	Median		5.7	2.6–8.9
	Range		0.4–30.3	
Follow-up (months, median)			5.7	2.2–9.3

Abbreviations: ICIs = Immune Checkpoint Inhibitors, PFS = Progression-Free Survival, OS = Overall Survival, Cri = Intracranial Complete Response, PRi = Intracranial Partial Response, SDi = Intracranial Disease Stabilization, PDi = Intracranial Disease Progression, PR = Partial Response, SD = Stable Disease, PD = Progressive Disease.

**Table 3:** Univariate and multivariate analysis using the Cox regression method to evaluate the effect of various parameters on survival outcome in the whole patient population (*n* = 280).

COX REGRESSION	PFS		OS	
Univariate Analysis	HR (95% CI)	*p*-Value	HR (95% CI)	*p*-Value
Age > 70 years old	0.858 (0.648–1.136)	0.285	0.794 (0.574–1.099)	0.164
Performance status = 2	1.517 (1.064–2.163)	0.021	2.132 (1.445–3.144)	<0.001
Non-squamous histology	0.999 (0.747–1.337)	0.997	1.065 (0.765–1.484)	0.708
ICI administration as 2nd or subsequent line of therapy	2.284 (1.519–3.436)	< 0.001	2.840 (1.665–4.846)	<0.001
Brain metastases	1.838 (1.314–2.569)	< 0.001	1.810 (1.230–2.662)	0.003
High disease burden *	1.746 (1.256–2.427)	0.001	1.961 (1.363–2.821)	<0.001
PD-L1 < 50%	2.083 (1.464–2.958)	< 0.001	1.915 (1.269–2.898)	0.002
**Multivariate Analysis**				
Performance status = 2	1.377 (0.882–2.149)	0.159	2.106 (1.269–3.497)	0.004
ICI administration as 2nd or subsequent line of therapy	1.672 (1.022–2.736)	0.041	2.898 (1.638–5.125)	<0.001
Brain metastases	2.265 (1.529–3.356)	< 0.001	1.577 (0.965–2.597)	0.069
High disease burden *	1.447 (0.909–2.303)	0.119	2.219 (1.380–3.579)	0.001
PD-L1 < 50%	1.709 (1.184–2.645)	0.005	1.390 (0.861–2.242)	0.178

Abbreviations: ICI = Immune Checkpoint Inhibitor, PFS = Progression-Free Survival, OS = Overall Survival, PD-L1 = Programmed Death-Ligand 1. * High disease burden is defined as >2 organs affected by metastatic disease.

**Table 4:** Univariate and multivariate analysis of the effect of the studied parameters on overall survival.

Variable	OVERALL SURVIVAL			
	Univariate		Multivariate	
	HR (CI 95%)	*p*-Value	HR (CI 95%)	*p*-Value
Age > 70 years old	1.723 (0.913–3.252)	0.093		
Performance status = 2	1.460 (0.739–2.883)	0.276		
Non-adenocarcinoma histology	2.150 (0.937–4.931)	0.071		
Second or subsequent line of ICI administration	1.628 (0.680–3.898)	0.274		
High disease burden *^1^	2.390 (1.161–4.922)	0.018	2.390 (1.161–4.922)	0.018
Non-primary CNS metastases *^2^	1.436 (0.760–2.711)	0.265		
PD-L1 extracranially ≥ 50%	1.021 (0.506–2.058)	0.954		

Abbreviations: ICIs = Immune Checkpoint Inhibitors, PD-L1 = Programmed Death-Ligand 1. *^1^ High disease burden is defined >2 organs affected by metastatic disease. *^2^ Non-primary CNS metastases are defined as the absence of brain metastases at diagnosis but develop later during the course of the disease.

## Supplementary Materials

The following are available online at https://www.mdpi.com/2072-6694/12/12/3707/s1, Figure S1: Local CNS treatment modalities that were administered to the patients. Figure S2: Effect of steroid administration on intracranial overall response rate A. In the whole patient population that was included for response assessment B. In the subgroup of patients with active BM. Figure S3: Effect of extracranial PD-L1 levels on intracranial overall response rate A. In the whole patient population that was included for response assessment B. In the subgroup of patients with active brain metastases. Figure S4: Effect of the presence of neurological symptoms attributed to brain metastases on A. PFS and B. OS in the subgroup of patients with brain metastases. Figure S5: Effect of disease burden on OS in patients with baseline brain metastases before ICI initiation. Figure S6: Study design. Table S1: Effect of clinical parameters on discordance between intracranial and extracranial disease stabilization rates in response to ICI administration. Table S2: Univariate and multivariate analysis on the effect of the studied patient parameters on intracranial time to progression after ICI administration.
