# Supplementary material for: Outcome of Patients with NSCLC and Brain Metastases Treated with Immune Checkpoint Inhibitors in a ‘Real-Life’ Setting
Source: Cancers (Basel). 2020 Dec 10;12(12):3707. doi: 10.3390/cancers12123707 (PMC7764720; doi:10.3390/cancers12123707)
Supplement: Supplementary file 1 [file cancers-12-03707-s001.pdf]

cancers

Supplementary materials: Outcome of Patients with NSCLC and Brain Metastases Treated with Immune Checkpoint Inhibitors in a 'Real-Life' Setting

Marcus Skribek, Konstantinos Rounis, Dimitrios Makrakis, Sofia Agelaki, Dimitris Mavroudis, Luigi De Petris, Simon Ekman and Georgios Tsakonas

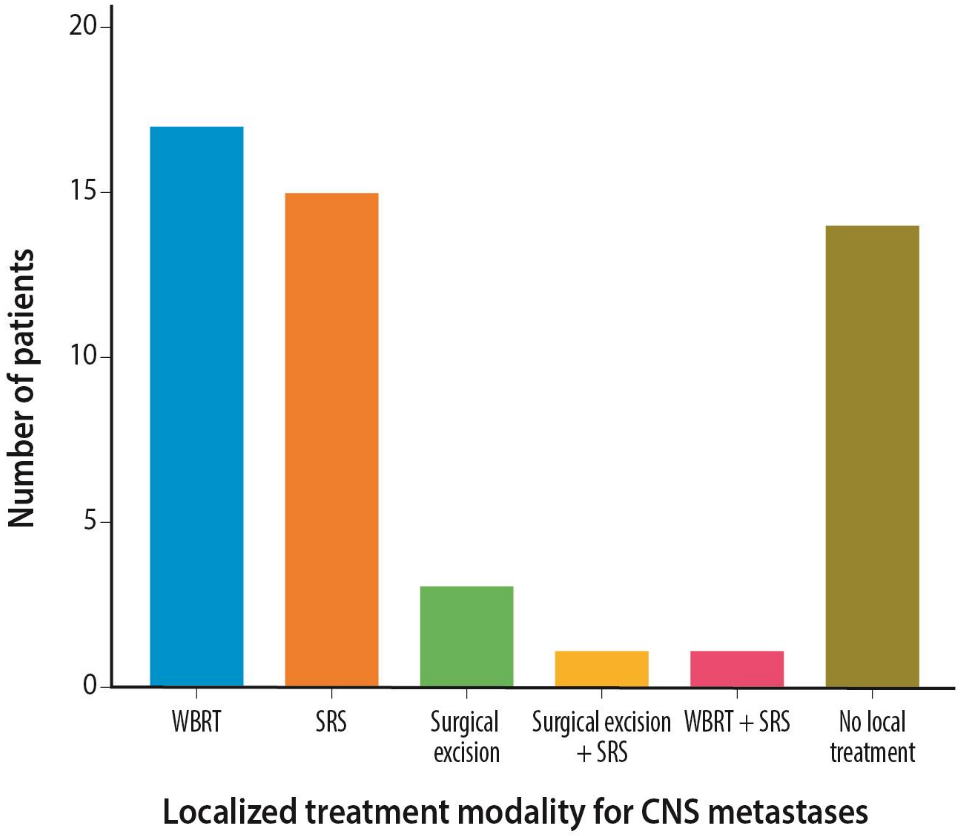

Figure S1. Local CNS treatment modalities that were administered to the patients. Abbreviations: WBRT = Whole Brain Radiotherapy, SRS = Stereotactic Radiosurgery.

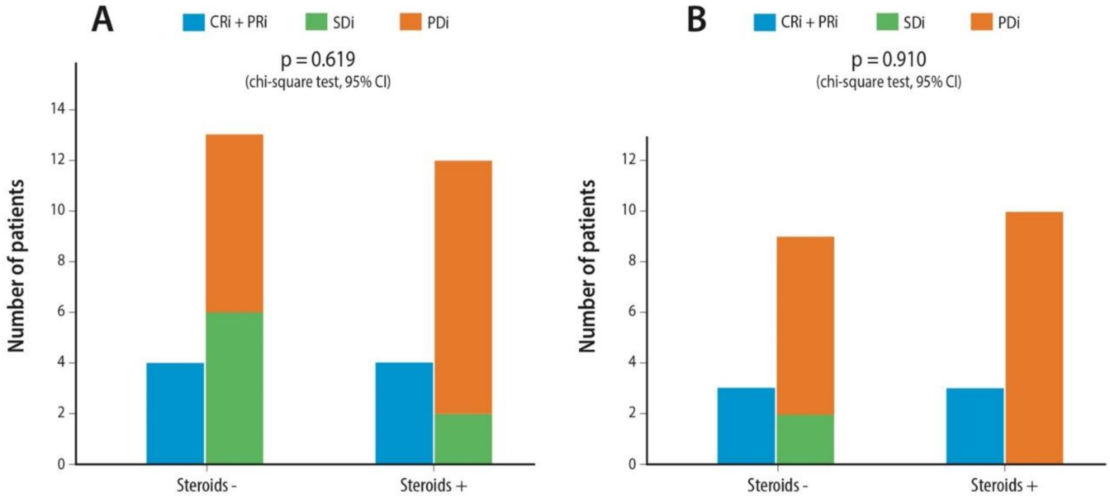

Figure S2. Effect of steroid administration on intracranial overall response rate **A.** In the whole patient population that was included for response assessment **B.** In the subgroup of patients with active BM. Abbreviations: CRi=Intracranial Complete Response, PRi=Intracranial Partial Response, SDi=Intracranial Stable Disease, PDi=Intracranial Progressive Disease.

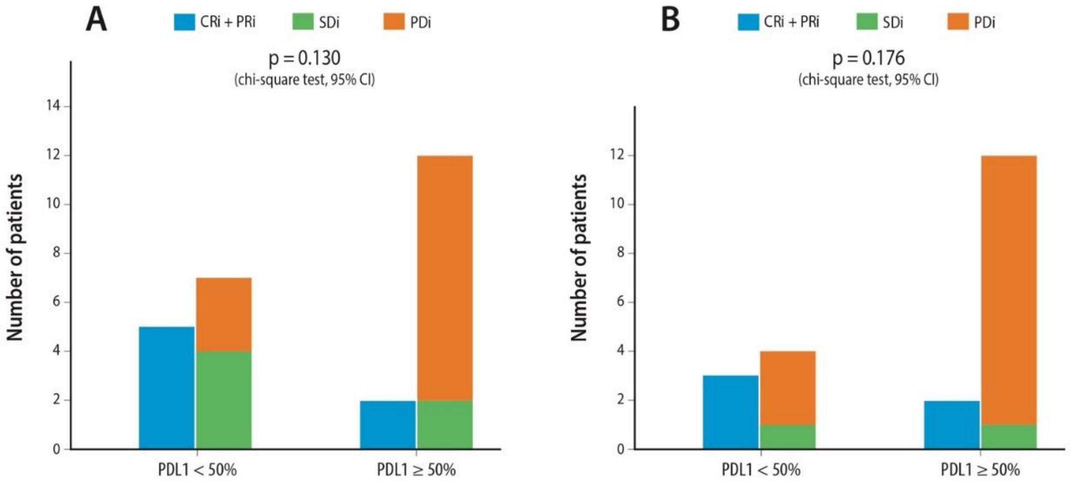

Figure S3. Effect of extracranial PD-L1 levels on intracranial overall response rate **A.** In the whole patient population that was included for response assessment **B.** In the subgroup of patients with active brain metastases.Abbreviations: CRi=Intracranial Complete Response, PRi=Intracranial Partial Response, SDi=Intracranial Stable Disease, PDi=Intracranial Progressive Disease, PD-L1=Programmed Death-Ligand 1.

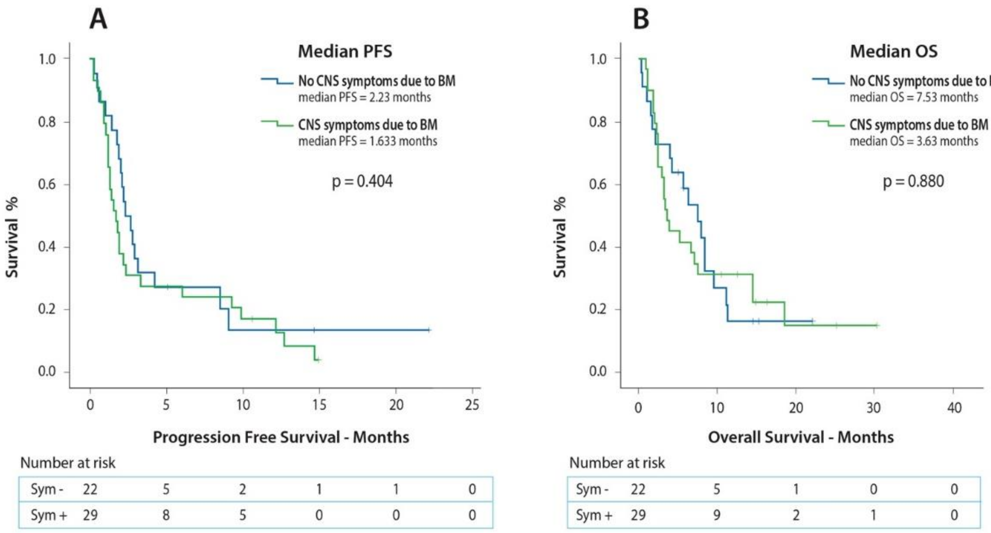

Figure S4. Effect of the presence of neurological symptoms attributed to brain metastases on **A.** PFS and **B.** OS in the subgroup of patients with brain metastases. Abbreviations: PFS=Progression-Free Survival, OS=Overall Survival, BM=Brain Metastases.

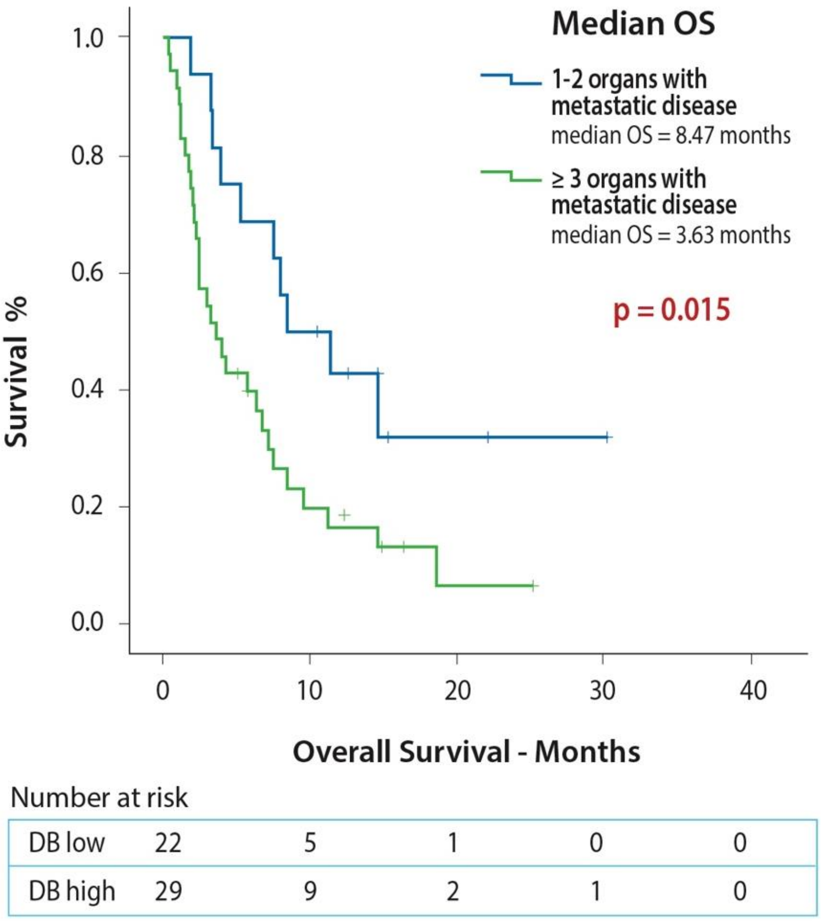

Figure S5. Effect of disease burden on OS in patients with baseline brain metastases before ICI initiation. Abbreviations: ICI=Immune Checkpoint Inhibitors, OS=Overall Survival, DB low=1-2 organs with metastatic disease, DB high= > 2 organs with metastatic disease.

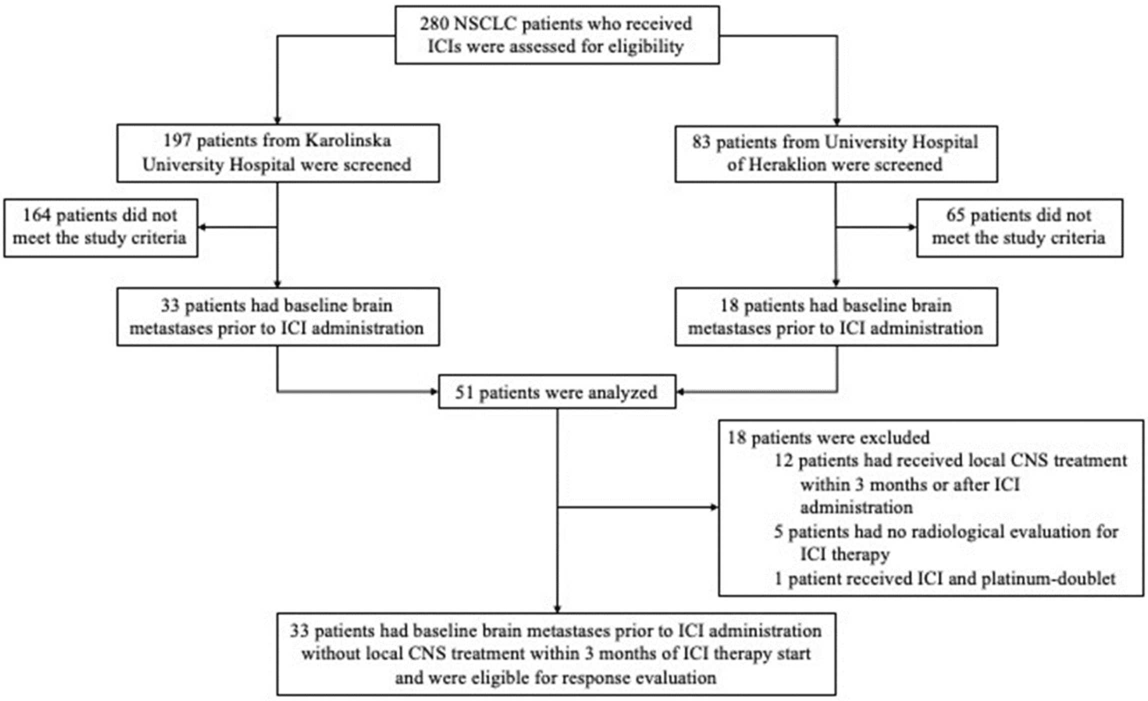

Figure S6. Study design. Abbreviations: ICIs=Immune Checkpoint Inhibitors.

Table S1. Effect of clinical parameters on discordance between intracranial and extracranial disease stabilization rates in response to ICI administration.

Variable		n	Discordance*[1]	No discordance*[1]	*p*-Value (chi-square test)
Age (years)	> 70	13	4	9	0.590
	≤ 70	20	8	12	
PS	0-1	25	11	14	0.107
	2	8	1	7	
Neurological symptoms*[2]	Yes	18	7	11	0.514
	No	15	5	10	
Steroid administration*[3]	Yes	16	5	11	0.410
	No	17	7	10	
Number of BM	> 3	12	3	9	0.305
	≤ 3	21	9	12	
Line of treatment of ICI administration	1st	5	1	4	0.409
	2nd or later	28	11	17	
Previous CNS radiotherapy	Yes	18	8	10	0.290
	No	15	4	11	
Primary BM	Primary	24	8	16	0.421
	Non-primary	9	4	5	
Active vs stable BM	Active	25	6	19	0.015
	Stable	8	6	2	

*1: Discordance among intracranial and extracranial disease stabilization (PR or SD) in response to ICI. *2: Neurological symptoms attributed to BM. *3: Steroid administration > 10 mg prednisolone equivalent for ≥ 10 days. Abbreviations: ICI=Immune Checkpoint Inhibitors, BM=Brain metastases, PR=Partial response, SD=Stable disease.

Table S2. Univariate and multivariate analysis on the effect of the studied patient parameters on intracranial time to progression after ICI administration.

Variable	INTRACRANIAL TIME TO PROGRESSION			
	Univariate analysis		*Multivariate analysis*	
	**HR (95% CI)**	***p*-Value**	**HR (95% CI)**	***p*-Value**
Age > 70 years old	2.045 (1.116–3.745)	0.021	1.567 (0.709–3.465)	0.267
Performance status = 2	1.871 (0.959–3.457)	0.071		
Female gender	1.261 (0.686–2.315)	0.455		
Smoker or former smoker	0.921 (0.383–2.187)	0.851		
Non-adenocarcinoma histology	1.979 (0.915–4.282)	0.083		
High disease burden*[1]	1.634 (0.849–3.142)	0.141		
> 3 CNS metastases	0.672 (0.355–1.271)	0.222		
Largest CNS metastasis ≥ 3 cm	0.694 (0.366–1.319)	0.265		
Symptoms from CNS metastases	1.300 (0.707–2.387)	0.398		
Previous radiotherapy modality (> 3 months before ICI administration)	0.463 (0.225–0.954)	0.037	1.469 (0.650–3.319)	0.355
Active CNS metastases	1.517 (0.663–3.471)	0.324		
ICI administration as second or subsequent line	0.861 (0.397–1.870)	0.706		
Non-primary CNS metastases	1.688 (0.987–3.060)	0.099		
Steroid administration > 10 mg prednisolone equivalent for ≥ 10 days	1.401 (0.764–2.588)	0.276		
PD-L1 levels extracranially ≥ 50%	2.376 (1.161–4.862)	0.018	1.702 (0.779–3.720)	0.182

*[1] High disease burden is defined as > 2 organs affected by metastatic disease. *[2] Non-primary CNS metastases are defined as the absence of brain metastases at diagnosis but develop later during the course of the disease. Abbreviations: ICIs=Immune Checkpoint Inhibitors, PD-L1=Programmed Death-Ligand 1.
